# Supplementary material for: Analysis of Graviresponse and Biological Effects of Vertical and Horizontal Clinorotation in Arabidopsis thaliana Root Tip
Source: Plants (Basel). 2021 Apr 9;10(4):734. doi: 10.3390/plants10040734 (PMC8070489; doi:10.3390/plants10040734)
Supplement: Supplementary file 1 [file plants-10-00734-s001.pdf]

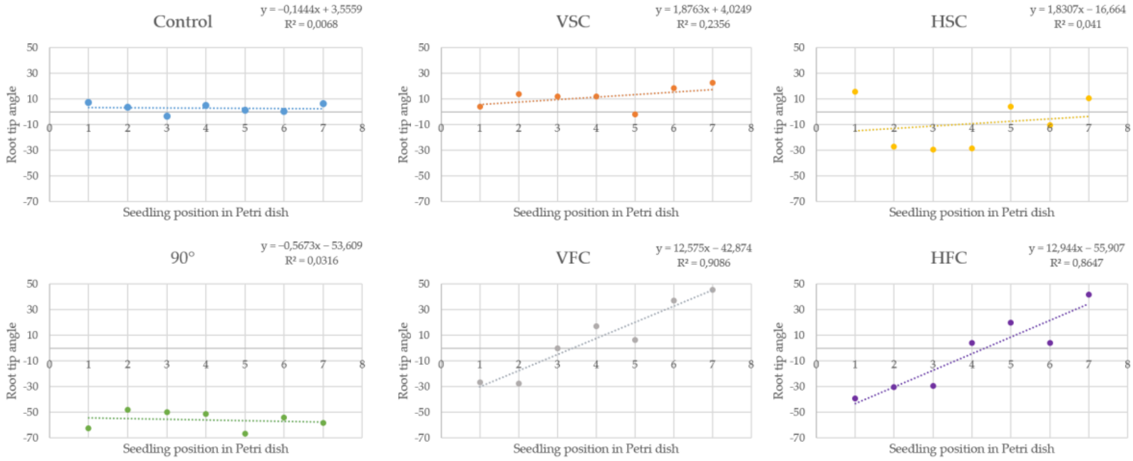

Figure S1. Relation between the distance from the rotation center and the angle of the root. Graph representation of average angle of seedlings (integral angular deviation) in the different positions on the plate in the different experimental conditions; Control; VSC, Vertical Slow Clinorotation; VFC, Vertical Fast Clinorotation; HSC, Horizontal Slow Clinorotation; HFC, Horizontal Fast Clinorotation; 90°, directional growth control.

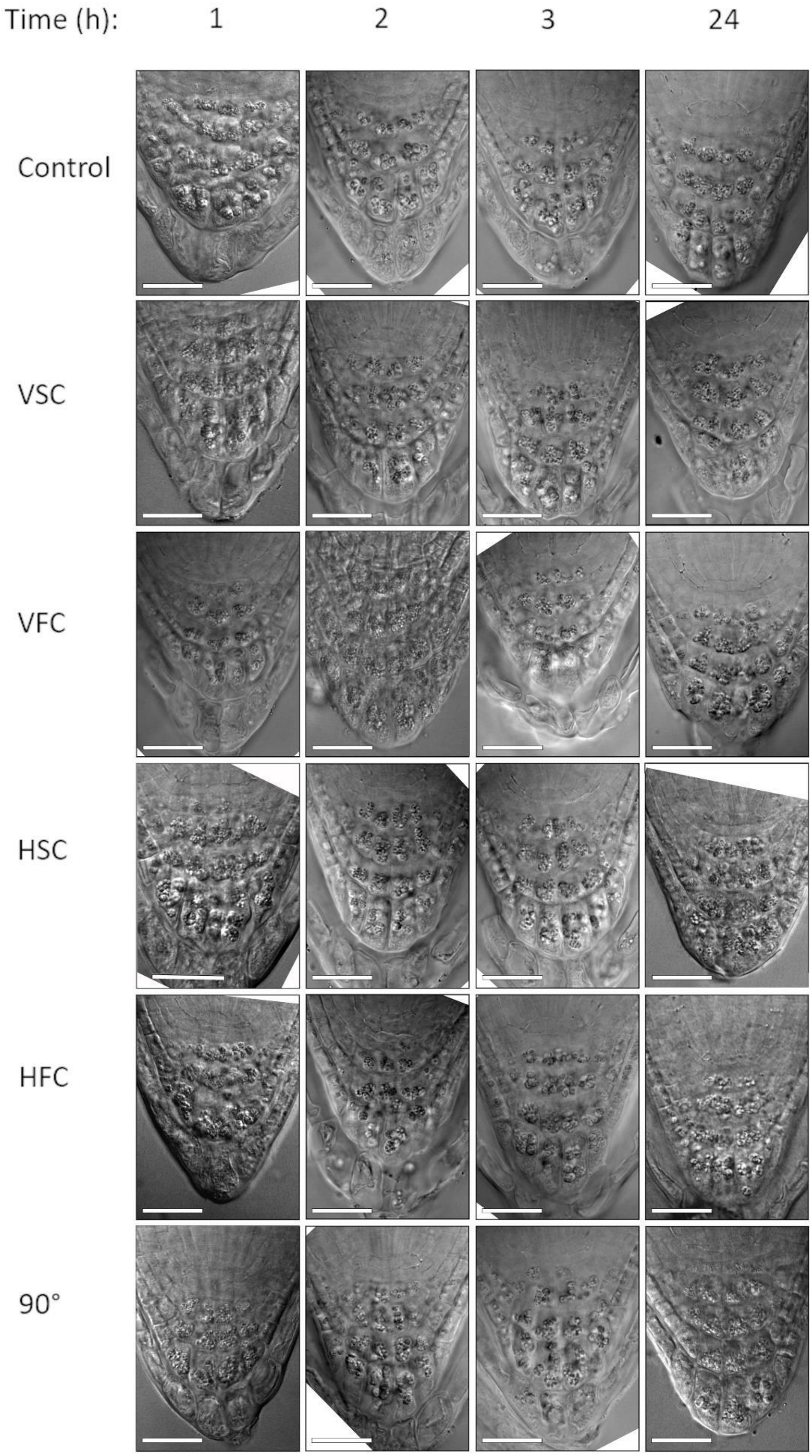

Figure S2. DIC images of columella. Images of statocytes in the different times and conditions: 1, 2, 3 and 24 h; Control, Vertical Slow Clinorotation (VSC), Vertical Fast Clinorotation (VFC), Horizontal Slow Clinorotation (HSC), Horizontal Fast Clinorotation (HFC) and directional growth control (90°). Scale bar represents 25 μm.

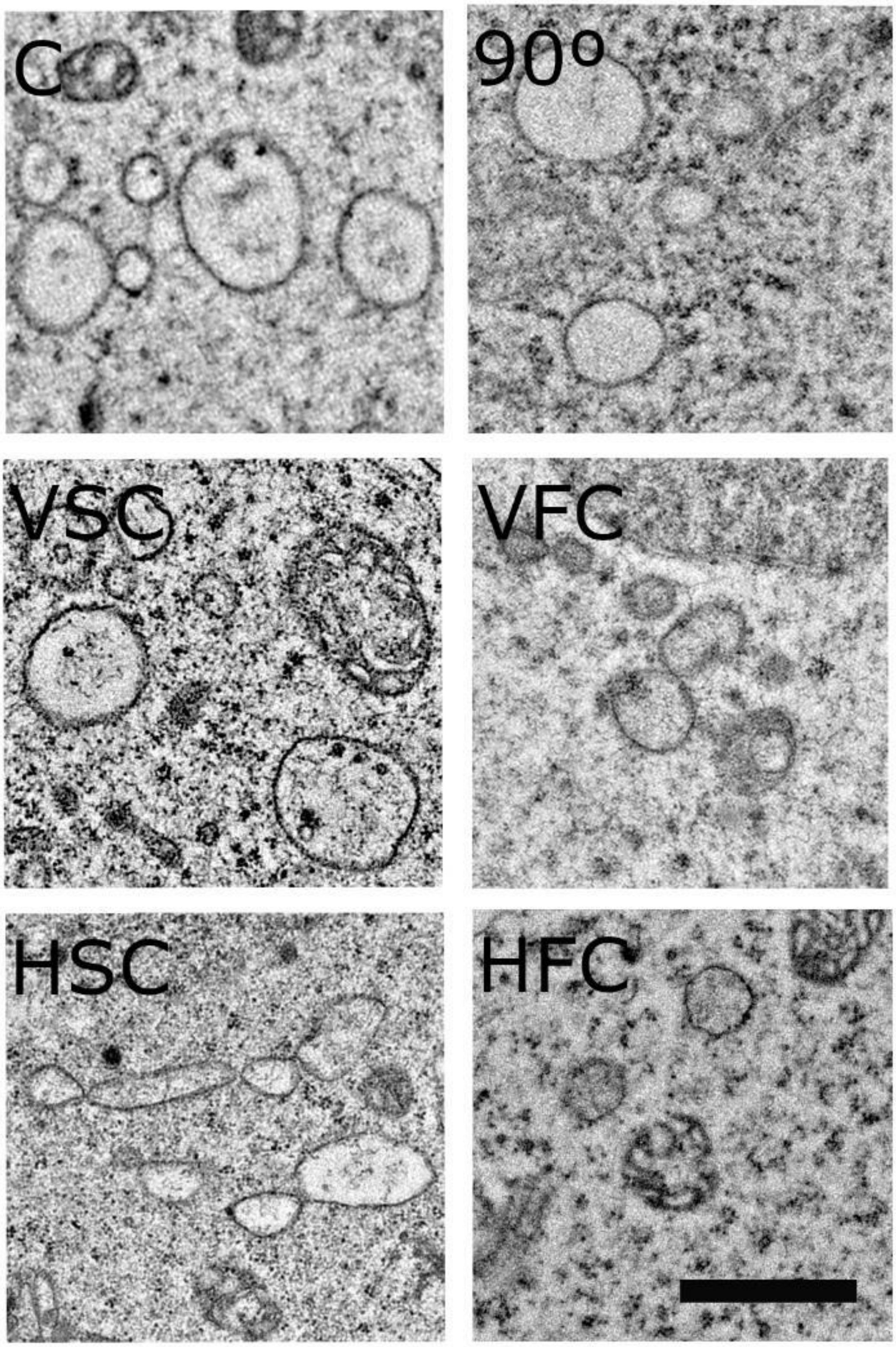

Figure S3. Detail of lysosomes in statocytes. Electron microscopy images of representative lysosomes in statocytes of S2 central columella tier in the different experimental conditions: Control (C), directional growth control (90°), Vertical Slow Clinorotation (VSC), Vertical Fast Clinorotation (VFC), Horizontal Slow Clinorotation (HSC) and Horizontal Fast Clinorotation (HFC) Scale bar represents 1 μm.

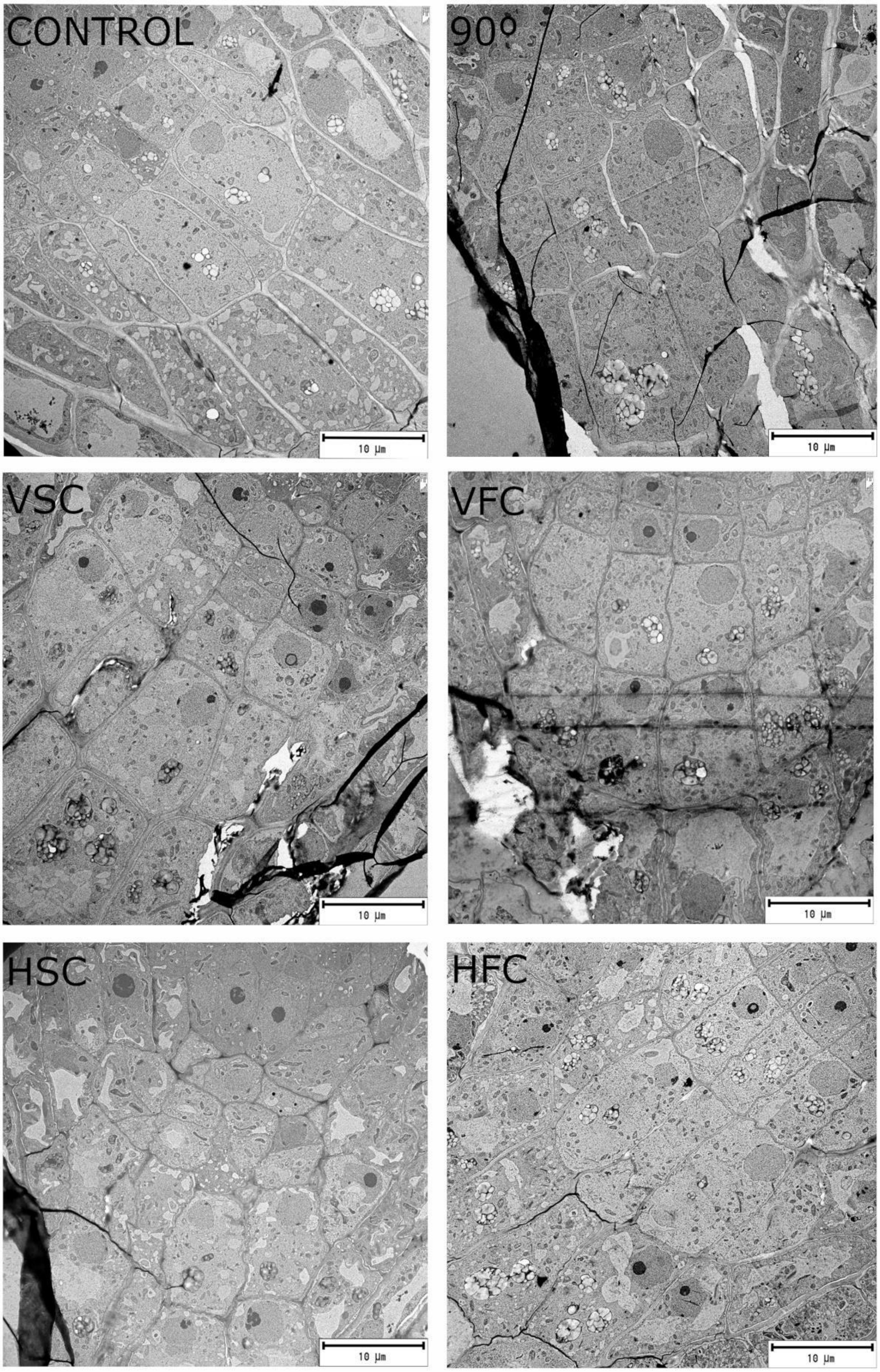

Figure S4. Columella structure. Electron microscopy images of whole columella in the different experimental conditions. Control (C), directional growth control (90°), Vertical Slow Clinorotation (VSC), Vertical Fast Clinorotation (VFC), Horizontal Slow Clinorotation (HSC) and Horizontal Fast Clinorotation (HFC Scale bar represents 10 µm.

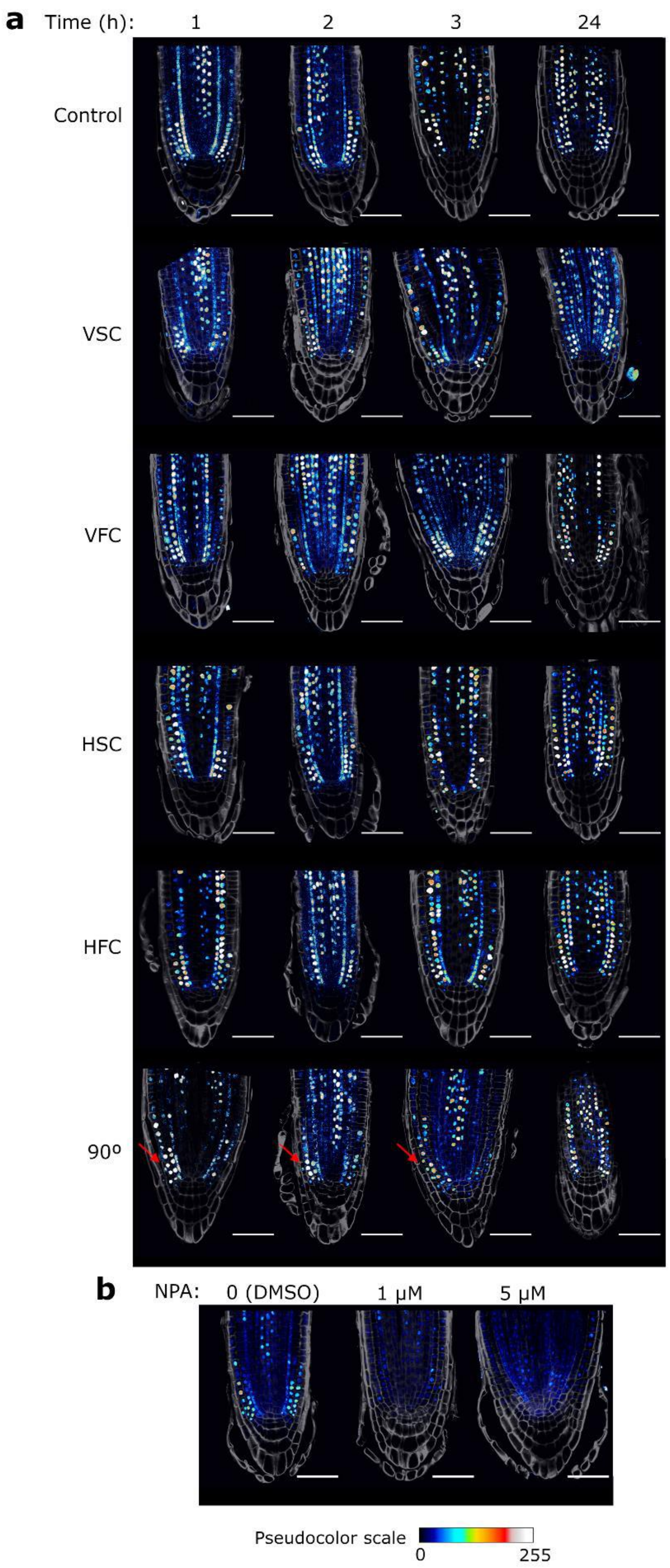

Figure S5. Auxin distribution with DII-Venus reporter seedlings. (**a**) Confocal microscope images of DII-Venus seedlings. Pseudocolor reflecting the intensity of the YFP signal was applied with the Lookup Table Royal Tool in the ImageJ software. Grey: cell wall staining with Renaissance SR2200. (**b**) DII-Venus seedlings grew for 5 days with 0 (DMSO), 1 or 5 µM NPA. Scale bar represents 50 µm.

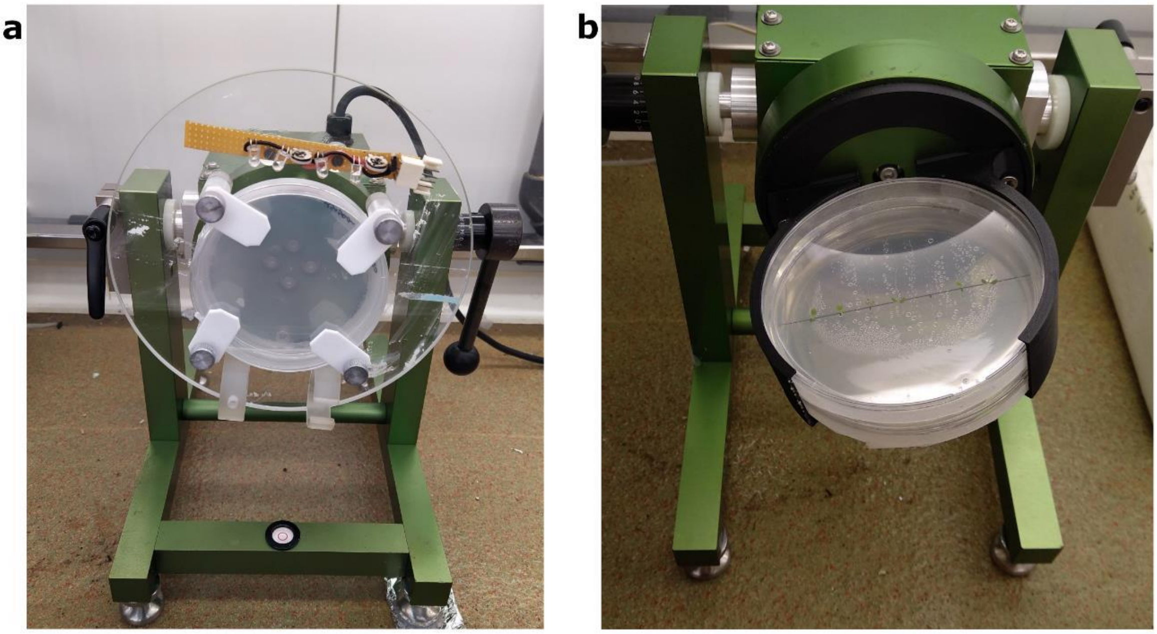

Figure S6. Clinostat used in this study. 2D-clinostat provided by Zero-Gravity Instrument Project (ZGIP, UNOOSA). (**a**) 2D-clinostat with vertical clinorotation adaptor (**b**) 2D-clinostat with horizontal clinorotation adaptor.
